# The professional resilience of mid-career GPs in the UK: a qualitative study

**DOI:** 10.3399/BJGP.2021.0230

**Published:** 2021-11-02

**Authors:** Lucy Martin, Almuth McDowall

**Affiliations:** Dudley, West Midlands.; Birkbeck University of London, London.

**Keywords:** general practice, general practitioner, delivery of health care, professional resilience, psychological resilience, social media

## Abstract

**Background:**

With a continued crisis of increasing workload and reduced workforce in general practice, supporting resilience is a key strategy for sustaining the profession into the future.

**Aim:**

How do GPs perceive professional resilience, and what workplace factors influence it?

**Design and setting:**

A UK-based qualitative study of the perspectives of GPs currently practicing in mainly urban locations across the UK with ≥5 years’ experience after completion of GP training.

**Method:**

Participants were recruited using convenience sampling, which included social media forums, and underwent semi-structured interviews undertaken in May and June 2020 (*n* = 27). Data were analysed using thematic analysis.

**Results:**

Participants offered definitions of and influences on resilience that largely fit with existing research, but in addition, may result in the perception that GPs are obstructive, or that resilience may be a ‘surface act’. GPs agree that the current focus on methods of improving resilience does support them, but there is significantly more to be done in this field. Social media activity aiming at GP support may be counterproductive. Reduction of clinical working hours is a common strategy to improve resilience.

**Conclusion:**

That GPs feel to improve resilience they need to work fewer clinical hours may have huge implications for a workforce already in crisis, and ultimately, for the health care of the UK population. Urgent research is needed to formulate a bespoke assessment for measuring GP resilience to assess potential interventions, and to identify GPs at risk of mental ill-health or leaving the profession.

## INTRODUCTION

General practice in the UK is facing a crisis.^1^^–^8 While patients remain highly satisfied with the care they receive, satisfaction with access to GPs has steadily declined.^9^ The number of full-time equivalent GPs per head of population is falling.^2^^,^^5^^,^^8^^,^^10^ However, a higher head count of GPs^11^ indicates a rise in less than full-time working patterns for clinical practice hours. Although the UK government has endeavoured to address workforce shortages, plans to recruit >5000 permanent GPs to practice by 2020 have resoundingly failed.^11^ National objectives have changed in their language from ‘extra GPs’ to ‘extra primary healthcare professionals’, perhaps acknowledging the lack of a ‘magic GP tree’.^12^^,^^13^

Undoubtedly, the demands of GP jobs continue to rise. Official measures of workload report a year-on-year rise since 2007.^12^^,^^14^ This may be significantly underestimated, as GPs report increased demands such as:

- loss of autonomy and control over workload;
- increasing administrative duties;
- transfer of work from secondary care as part of the drive to manage more conditions in the community;
- regulatory activity; and
- a complaints-driven culture.

In addition, GPs report reduced work resources, such as insufficient staff recruitment and retention, and time pressures.^1^^,^^4^^,^^5^^,^^12^^,^^15^^–^18 GP mental ill-health and work-related stress is also on the increase.^6^^,^^19^ Proxy measures for these factors include burnout,^20^ which remains high in medical professionals in general and GPs in particular.^21^^–^24

Government key ambitions for primary care from 2016–2021 are ‘investment, workforce, workload, infrastructure, and care redesign’.^12^^,^^25^ While offering a comprehensive national mental health support plan to doctors^13^^,^^25^ an opportunity has been missed to address potential root causes of the problem, rather than offering solutions to those already adversely affected. Moreover, there is no exact forecast of the longer-term challenges that the COVID-19 pandemic will bring to primary care.

Given the crucial role of the GP in health care, delivery resilience in the profession is of growing interest and features clearly in national strategies.^12^^,^^25^ This suggests that by becoming a more resilient GP or practice, the demands at work can be overcome. There has been development of specific programmes for GPs with the aim of improving the resilience of the individual doctor or the entire practice; however, evaluative research is scarce.^5^^,^^26^^,^^27^ Previously, the focus has been on attracting more new recruits to the profession, enabling return to practice, and preventing early retirement. But GPs in mid-career have not attracted any such attention. This group of practicing clinicians, defined as having ≥5 years in practice but with no specific retirement plans, are the focus of this study.

**Table:** How this fits in

In this study, GPs identify ‘good’ mid-career resilience and protective factors such as social support, which concurs with existing research. The novel contribution is the identification of clear factors that reduce the professional resilience of GPs in the UK. A GP with strong resilience may exhibit obstructive work behaviours and surface acting to demonstrate resilience. Resilience has become another work task for GPs. Social media, despite being intended to be supportive, can act as a drain on resilience.

While the concept of psychological resilience is well researched, the precise definition remains unclear. There is broad agreement that resilience is a process and a dynamic phenomenon affected by environmental, personality, cultural, sociological, and biological factors.^28^ Indeed, context is important, as behaviours and outcomes that are conducive in some contexts may be counterproductive or not effective in others.^29^

Although limited research about resilience in UK medical or GP-specific contexts exists, the phenomenon remains ill-defined.^4^^,^^21^^,^^22^^,^^26^^,^^27^^,^^30^^–^32 There is no agreement about how resilience should be measured, the effect of any interventions,^5^^,^^33^^,^^34^ or how resilience affects patient care.

A qualitative approach was used to generate understanding on the central research questions, concentrating on GPs with some years of experience in work. They were chosen because they are a large group without specific strategies for retention in the profession. They have the capacity to speak both from their own experience and about the sociopolitical context of UK general practice. They have some understanding of their own phase of professional resilience, what factors influence it, and how that view may differ from NHS England’s or from existing research on psychological resilience. Therefore, the overall research question was: how do GPs perceive professional resilience, and what workplace factors influence it?

## METHOD

### Semi-structured interview design and approach

Semi-structured interviews were undertaken in May and June 2020, which coincided with the COVID-19 pandemic. Owing to restrictions on physical contact, interviews were conducted online via video calls, although telephone calls were also offered to include participants who lacked the appropriate technology. Previous research suggests that the virtual mode of interview makes little difference to the final data collected.^35^

An interview template was devised through an iterative process and piloted with three GP volunteers to refine content and to balance free discussion with a consistent structure (see Supplementary Appendix S1 for details). To build rapport, the semi-structured interviews began with open questions. The further schedule asked GP participants about their career to date, current work situation, thoughts on definitions of and influences on professional, organisational, and personal resilience, the current approaches to improvement of resilience, and coping mechanisms for work-related challenges. All interviews were conducted by the lead author. They were all audiorecorded, and lasted between 24 and 100 min (mean 49 min, median 43 min). Only three interviews were >65 min in duration. All interviews were fully transcribed.

### Participants and recruitment

Following a favourable ethical opinion from the researchers’ institution, recruitment took place via several methods to obtain a purposive sample of working GPs of varied demographics. The information sheet and consent form were uploaded to an online survey platform. Primary Care Networks (PCNs) local to the research were sent the survey link, and GP colleagues disseminated these among their own local networks. The link was also shared through social media platforms (Twitter and private GP groups on Facebook). Once a participant had completed the online form and given consent to participate, they were contacted to arrange an interview.

For inclusion in the study, GPs had to have been working in the UK NHS within the previous 12 months and have ≥5 years’ experience after completion of GP training. Participants received no financial reimbursement for involvement. In total, 27 GPs took part. As detailed in Table 1, they had varied working hours, partners, were locums and salaried GPs, and of mixed ages and sex. As detailed in Table 2, they were from different geographical locations. Eight participants were from the same clinical commissioning group (CCG) area for convenience, but from five of six PCNs in that CCG. Interviews took place during May and June 2020, during the first wave of the COVID-19 pandemic in the UK.

**Table 1.** Participant demographics (*N* = 27)

**Demographic**	***n* (%)**
**Age, years**	
20–29	0 (0)
30–39	4 (14.8)
40–49	15 (55.6)
50–59	8 (29.6)
60–69	0 (0)

**Sex**	
Female	19 (70.4)
Male	8 (29.6)

**Type of GP**	
Partner	21 (77.8)
Salaried GP	4 (14.8)
Locum	2 (7.4)

**Pattern of work**	
Practice-only GP	10 (37.0)
Portfolio GP	17 (63.0)

**Years in practice**	
5–10	4 (14.8)
11–15	9 (33.3)
16–20	5 (18.5)
21–25	4 (14.8)
26–30	4 (14.8)
31–35	1 (3.7)

**Table 2.** Geographical location of participants work (*N* = 27)

**Location of participants work**	***n* (%)**
**Country**	
England	25 (92.6)
Scotland	1 (3.7)
Wales	1 (3.7)
Northern Ireland	0 (0)

**CCG**	
Airedale	1 (3.7)
Barnsley	2 (7.4)
Bassetlaw	1 (3.7)
Birmingham and Solihull	1 (3.7)
Coventry	1 (3.7)
Dudley	8 (29.6)
Herts Valleys	2 (7.4)
North Somerset	1 (3.7)
Nottinghamshire	1 (3.7)
Oxford City	1 (3.7)
Powis	1 (3.7)
Salford	1 (3.7)
South Warwickshire	1 (3.7)
Southampton	1 (3.7)
Stockport	2 (7.4)
Tayside Health Board	1 (3.7)
Trafford	1 (3.7)

*CCG = clinical commissioning group.*

### Data analysis

The analysis was undertaken from a constructivist epistemology, whereby close attention was paid to the meaning of any concept as construed by the participants, while bearing in mind specific aims about participants’ conceptualisation of resilience. The data were analysed with thematic analysis aligned to the six-step protocol by Braun and Clarke.^36^ The initial prescriptive coding of each transcript by the lead author was then reviewed and interpreted further to reveal the underlying thoughts and ideas, which were grouped together as themes. Relevant and pertinent data were recorded from each interview in an iterative process; saturation (where no new themes were coded) was reached after 23 transcripts.

## RESULTS

### Findings

A summary of the themes generated by this research is detailed in Box 1. The findings outline the definition of resilience and what GPs identify as positive and negative influences on their resilience, and serve to add more detail to the existing literature. The themes are illustrated with pertinent quotes.

**Box 1.** Summary of themes (codes) identified

**Theme**	**Subcodes**	**Explanation**
Definitions/origins	Personal resilience definition	Difference in definitions highlights tension between organisational and individual definitions — so will vary by personal experience and also by how expectations are spelled out
	Professional resilience definition	
	Organisational resilience definition	
	Use of the term in multiple contexts at work	
	Resilience as part of personality/individual characteristics	

Costs of endeavouring high resilience	Saying no/obstructive behaviours	Exemplifies a conundrum — resource-preserving strategies for individuals (for example, cutting clinical hours) may in turn deplete resources for patient care
	Effect on colleagues	
	Effect on patients — reduced access, reduced continuity	
	Consumption of resource — time and financial	
	Human cost to GP	

Benefits of high resilience	Organisational benefit — improved working conditions, staff retention	Preserving resilience links to beneficial individual, organisational, and patient outcomes and serves to preserve capacity in a stretched system
	Improvement of outcomes for patients	
	Personal benefit — improved mental health, reduced burnout, performing well at work, job satisfaction, career progression, being able to ask for help, a reserve capacity for the future at work	

Relationships between personal and professional resilience	Professional resilience can be learned	Need to recognise the work–home and work–person interface; a holistic consideration is needed
	Both are interlinked	
	Both are separate	
	Can be different at home and at work	

Enhancers of resilience at work	Good communication	The data corroborate that social factors *in situ* are crucial; no mention of formal training
	Mentoring/support from colleagues	
	Accepting failure is part of work	
	Good team around you	
	Socialising in and out of work	

Reducers of resilience at work	Burden of fear of complaints/litigation	This theme illustrates how resilience could be enhanced by focusing GPs on meaning of their work, and putting in place administrative systems to combat ‘administration overload’
	COVID-19 working	
	Organisational change	
	Overburdensome regulator	
	Appraisal and revalidation process	
	Workload/work intensification/time pressures	
	Financial/partnership/business/estates pressures	
	Poor work relationships	
	Meeting patient demand	
	Burdensome IT systems	
	Workforce issues	
	Isolation at work	

Enhancers of resilience outside of work	Hobbies	Finding balance with activities outside work is crucial; which of these are most effective depends on the individual
	Mental health	
	Good sleep	
	Exercise	
	Coping with personal adverse circumstances	
	Family support/life partner support	
	Friends (medical and non-medical)	
	Work–life balance	
	Pet ownership	
	Religious faith	

Reducers of resilience outside of work	Perfectionism	Both external events and influences, but also personal tendencies such as perfectionism, need to be recognised
	Social media	
	Chronic ill-health	
	Challenge in personal life	
	Life events	
	‘Always-on’ culture	

#### Definitions of resilience

Most of the GP participants admitted that they felt unsure about the definition of resilience but acknowledged its current wide use in the NHS:

*‘I always think the term resilience is kind of a difficult thing to … try and pinpoint what it actually means.’*

(GP15, female [F], aged 50 years, 21 years in practice [YP], salaried GP [S], urban location [U])

Most participants reported that resilience was part of what defined them as a GP and as a professional. Furthermore, resilience was also about having an identity outside of being a GP, and encompassed personal resilience:

*‘I guess it is all to do with the persona of being a doctor and the profession as a whole.’*

(GP8, F, aged 40 years, 12 YP, Partner [P], U)

*‘Professional resilience is when I am feeling overwhelmed, I am not sure what I am doing and I need help … I think you have to deal with it in a way that remains professional.’*

(GP25, F, aged 44 years, 11 YP, P, U)

*‘For personal resilience it is important for me to have something else that I do, something that it is important, so it doesn’t feel like all I do is be a GP.’*

(GP12, F, aged 55 years, 27 YP, S, U)

Participants described the dynamic process of resilience, explaining how they found ways to manage their personal life and life events, adapt to those changes, and maintain their ability to carry on working effectively. Despite adverse life events such as bereavement, infertility, physical and mental illness, and serious conflict at work, all participants were working in a clinical capacity as well as in other roles, all while maintaining their personal lives outside medicine. Every participant talked about how they had felt different levels of resilience at different times in their lives and careers, and how they managed to continue working despite low resilience:

*‘When I had had my baby I* [came] *back straight in and joined as partner. I found that first 6 months with a 10-months-old at home and my older one and, I felt that over that 6 months I really struggled with resilience, I didn*’ *t have any. Because all of my energy was spent trying to prove that I was not lazy, because no one had worked here part-time before.’*

(GP17, F, aged 42 years, 9 YP, P, U)

*‘Resigning from a partnership is one of the biggest, scariest steps I have ever taken in my career. I thought once I have done that I can do most challenges, so I think it has helped my resilience really.’*

(GP22, F, aged 47 years, 15 YP, S, U)

Participants noted that resilience meant feeling mentally and physically well and happy at work, which enhanced the experience of caring for patients. This entailed a high level of job satisfaction and enjoyment; a feeling they had done their best, that they were doing a good enough job or performing well in a difficult job, and feeling stable in their working environment:

*‘Professional resilience is you keep* [GPs] *happy and engaged which makes them better doctors as well.’*

(GP10, male [M], aged 49 years, 20 YP, P, U)

Crucially, resilience was about a positive sense of thriving at work, and more than just getting by:

*‘I think back to when I was sort of stressed and burnt out I was not enjoying my job, not enjoying life full stop. And I did not feel like I was being successful in anything that I was doing, it was very much about survival rather than thriving. And I think, for me resilience is being able to thrive.’*

(GP14, F, aged 37 years, 6 YP, Locum [L], U)

Most participants felt that resilience equalled coping and not letting the workload overwhelm them. They were able to ask for help and deal with the rigours of supporting patients, colleagues, and staff, and moving forward in their careers. Participants described the importance of a reserve capacity to deal with future adversity that may or may not occur, as well as a capacity to be able to reflect on their work, recognise that things need to change, either personally or organisationally, and to be able to action that change:

*‘If you are resilient, when you have additional stresses that come in professionally you have the capacity there to cope with those.’*

(GP14, F, aged 37 years, 6 YP, L, U)

Importantly, coping with pressure also extended to actively supporting colleagues and trainees:

*‘* [Resilience is] *coping with all the difficult things like seeing what our patients are going through and trying to be there for them, and trying to make sure you are doing your best with colleagues and trainees to support* [them] *as well.’*

(GP16, F, aged 56 years, 28 YP, P, U)

#### Influences on resilience

Participants had different notions about how to improve their resilience in work. Some influences were related to the organisations in which they worked, such as a well-run practice, good staff, and stable finances.

Other influences included their relationships in and out of work, good leadership in the practice, clinical competence, good communication, and sharing of responsibility. They also identified shared social time with colleagues (particularly through breaks from work and getting together with colleagues during the working day) and high levels of continuity of care with patients as protective influences. In addition, having support and hobbies or interests outside of work was also significant.

Strongly reflected in most of the interviews was that external factors reduced resilience. Top-down change imposed by government and excessive, regular changes to the GP contract, excessive regulation, an overly target-driven culture, a hostile public perception of the profession, and a perceived lack of support from the Royal College of General Practitioners were all identified as significant negative influences, which left GPs with a sense of having lost their voice and identity:

*‘I think there is too much focus in organisations around delivery of targets and patients experience, maybe at the expense of staff. I do feel a little bit like many of us in the NHS are cannon fodder.’*

(GP18, M, aged 50 years, 13 YP, P, U)

*‘Primary care is really, really difficult, but also it’s the constant denigration of our profession — really, that doesn*’ *t help.’*

(GP2, F, aged 42 years, 12 YP, P, U)

*‘In the background I feel there is no great voice for general practice in the UK, I feel the Royal College of GPs are completely anonymous.’*

(GP6, M, aged 47 years, 18 YP, P, U)

Organisational factors such as long-term absences of GPs and other staff, problems with recruitment, personal and partnership financial difficulties, complex pension and tax issues, poorly functioning IT systems, rising practice list sizes, increasing patient demand, and a need to provide high levels of patient satisfaction were all identified as factors that reduce resilience at work. Work intensification was also highlighted by most participants, which includes aspects such as long working hours, few breaks, time pressures, and constant interruptions as being the norm for a working day:

*‘I think the constant decision making is what I struggle with. I get home from work with a splitting tension headache … it is just the pace of the day.’*

(GP23, F, aged 36 years, 8 YP, P, U)

This was highlighted further by the timing of the research: participants reflected that the way COVID-19 had affected their work was not yet mature. Working practices were changing but there was a sense that it would not reduce the work intensification for GPs.

Personal factors contributed to reduced resilience: life events, a poor work–life balance, caring responsibilities outside of work, financial outgoings, isolation at work, a tendency to be self-critical, and the emotional burden of absorbing patient distress repeatedly, day after day.

#### Being obstructive and surface acting

Much of the above influences reflect what has been noted in previous research on resilience in medicine. However, this research also suggests that resilience may mean that GPs feel they need to deflect or be more obstructive towards additional work.

Many participants reported refusing to do certain aspects of work (work outside the General Medical Services contract, or work passed down from secondary care) for the purpose of self-protection, with patients being impacted negatively as a result. The participants also acknowledged how this might impact on practice colleagues, but also it may change how GPs are perceived by colleagues or the public:

*‘People are finding the only way they can cope is to just start saying no to things. But I do worry that that’s going to undermine the kind of, essence of general practice, turn it into something that it hasn*’ *t been.’*

(GP8, F, aged 40 years, 12 YP, P, U)

This research suggests resilience may be an act, either at the surface or deep, with potential long-term consequences.^37^ Some of the participants explained surface acting, where resilience is demonstrating the capacity to remain stoical in the face of an ever-increasing workload and patient demand:

*‘We should just get on with everything and take it as it comes.* [Resilience] *seems to be another word for get on with it all and keep amassing, it doesn*’*t matter how much there is; just grin and bear it really.’*

(GP11, F, aged 49 years, 20 YP, L, U)

Resilience conceptualised in this way is to endure hardship without showing one’s emotions or complaining. Being resilient was sometimes linked with appearing uncaring; highly resilient GPs could appear to work efficiently without getting too emotionally invested:

*‘I know some GPs who are incredibly caring; they make great GPs but it is not good for their resilience and at the other end you meet* [GPs] *who couldn*’ *t care less but they go home happy every night.’*

(GP9, F, aged 43 years, 16 YP, P, U)

#### The counterproductive effects of social media

The rise of social media forums for GPs has perhaps provided a virtual replacement of the old social spaces colleagues would inhabit for support, for example, the doctors’ mess.^38^ Social media aiming at supporting GPs may be counterproductive; many participants identified that social media felt difficult — even hostile — at times, and reduced their resilience at work:

*‘Sometimes … it seems a bit bullish and very obstructive and if I am not in a good place and I read through some of* [the Facebook group] *it does make me feel very negative about the profession.’*

(GP11, F, aged 49 years, 20 YP, L, U)

#### Is reducing hours the best mechanism?

GPs agreed that a means for achieving good resilience was, for many of them, to reduce their clinical working hours. Many had already reduced their clinical work or planned to do so in the future, or reduce clinical hours still further. Participants described the need to reduce clinical hours to be able to make it through their working life to retirement. Many GPs had taken on non-clinical work (such as portfolio work: education, leadership roles, PCN roles, charity roles, coaching, and private enterprise) to be able to maintain their income while reducing clinical hours. This was important, as many had financial commitments that needed maintaining (for example, mortgage, school fees, and so on). Taking on other roles outside of their clinical GP work was interesting and valuable for their own personal development and job satisfaction, and was remunerated. This variety (having something to do other than clinical medicine) was extremely important for their resilience at work. Every participant discussed how this improved their professional resilience:

*‘You know, the thought of working eight or nine clinical sessions these days, and doing nothing else, is just, I don’t know how I could manage that.’*

(GP1, M, aged 47 years, 18 YP, P, U)

*‘Senior partners used to do 5 days a week, nine sessions full time … you are working too much and you need to reduce that. It’s a different job intensity-wise since they started.’*

(GP7, F, aged 38 years, 9 YP, P, U)

## DISCUSSION

### Summary

Resilience in GPs could be presented as a model of job demands and resources;^39^ all jobs have characteristics that can be classified as either demands or resources. As the participants of this research explain, their resilience is negatively affected by job demands (such as workload, poor staffing levels, and poor facilities) and moderated by resources (such as colleague support, a competent practice manager, staying mentally and physically well, and a portfolio career), which chimes with mainstream research on resilience. This study’s data also highlight contextually driven strategies to maintain professional resilience; that is, the capacity to sustain the GP role over time.

GPs describe that they are reducing clinical work as a method of improving their resilience. Almost all participants had either reduced or were considering reducing their clinical work to enable them to maintain the rest of their careers. This strategy may make the individual GP more resilient, but could be making the organisation (practice or the NHS as a whole) less resilient. Most of the GPs were not opting to utilise hours for more leisure time, but for alternative non-clinical work or clinical work in a different setting to the GP surgery. This was not only a mechanism to sustain income, but also a means of keeping motivated. Such strategies may be linked to a relatively flat GP career structure that may lack opportunities to move into more senior positions or to take on specialised tasks.

This research also suggests that there might be unintended consequences from endeavouring to enact resilience. Strategies include deflecting additional work or appearing obstructive to demands for the purposes of self-protection, but at the possible expense of colleague and public perception. Some participants admitted that this was essential to preserve the self, yet was contrary to the image of a GP who is always working in the best interests of their patients.

Another facet was that resilience may be based on surface acting as part of the professional persona or duty of a doctor.^37^ GPs felt it important to demonstrate resilience, outwardly disguising their exhaustion and negative emotions. The conclusion is that there is potential shame and stigma about being seen as not resilient or weak in some way. Has having low resilience become synonymous with the failure of the individual GP rather than an acceptance that GP work demands are unmanageable for most?^17^ Participants felt that resilience has become yet another responsibility of the individual GP; another task that needed time, energy, resources, and personal investment by the GP. The long-term costs of pretence may, however, be significant, and could lead to accelerated workforce losses.

Social media has developed methods of mutual support with the evolution of groups of GPs on Facebook and other platforms, but may be counterproductive to professional resilience. GP participants reported that they made them feel unhappy and ‘unresilient’. Social media could be another source of pressure for GPs; a standard of ‘online’ resilience that GPs feel they should be working towards, and feel like they have failed if they do not meet it.

### Strengths and limitations

It is acknowledged that conducting a qualitative study in the early months of a world pandemic may influence opinions on resilience. However, a strength of this study is that data were collected from participants from a range of demographics, practices, and roles, albeit mostly working in England. The sample size and in-depth interview approach meant that data saturation was reached. It cannot be discounted that the participants may have taken part because of a history of difficulties with resilience, constituting a potential self-selection bias. Conducting the study with a single researcher interviewing participants and coding data could have subjected the data to both observation and confirmation bias. This was mitigated by the involvement in the analysis of a second author who does not work in a healthcare context. It may not be possible to extrapolate findings across the GP population, as GPs who feel very resilient and happy with their clinical work may have had little interest in taking part.

### Comparison with existing literature

Existing research on psychological resilience centres on a dynamic process.^40^ Resilience is affected by environmental, personality, cultural, sociological, and biological factors, but the person’s context is important and will change the definition. GPs’ views on professional resilience did not focus on this, but participants were able to identify what good resilience was. They were, however, less likely to elaborate on any of the methods of working towards good resilience. Furthermore, the data show that a holistic perspective on resilience that also recognises the work–life interface is needed. GPs agreed that current support offers from NHS England to some extent support improving resilience, but there is significantly more to be done in this field. The GP Forward View details a mentoring and support programme for resilience in general practice.^12^ The GP participants felt this was important. Many of the participants acknowledged that taking part in mentoring or other support programmes or therapy has been helpful for their resilience at work. They also commented that undertaking this could be perceived as a weakness by others, or that GPs most in need of support do not have the time to access it.

### Implications for research and practice

Specific interventions for resilience boosting may help the GP workforce. Box 1 illustrates that an approach that is holistic, wide-ranging, but also person-focused is warranted, one which goes beyond the immediate realm of work.

However, the barriers to accessing such approaches and the stigma of doing so needs to be overcome before GPs can utilise these routinely as part of their normal practice. GPs clearly identify many issues with workload and work intensification as well as patient demand and expectation, which need to be addressed urgently.

Professional resilience in mid-career GPs is a widely discussed yet poorly understood concept. Focus on how this can be measured would enable research to more accurately assess the effectiveness of a range of resilience interventions that are useful for GPs to undertake at different points in their careers, to establish who is at risk of mental ill-health or leaving the profession.

If GPs perceive that being seemingly obstructive is the only way to reduce work demand, this indicates that workload has reached unmanageable proportions. If workload and other job demands are not addressed urgently this is likely to worsen, fuelled by the GP social media groups and recent hostility towards GPs in mainstream media, which in turn could significantly further damage resilience.

The potential long-term costs of ‘resilience surface acting’, where GPs give the perception of doing well or coping yet may feel too stigmatised or too time-poor to access resilience interventions, are a particular concern. GPs appear to be resolving this issue by reducing their clinical working hours. The cumulative effects of fewer mid-career GP hours worked combined with increased retirement and reduced intake into the profession from newly qualified doctors are potentially grave concerns for the stability of the overall GP workforce, the NHS, and the health of the UK population as a whole.

Urgent consideration must be given to this by GP leaders, NHS England, and the Department of Health and Social Care to understand how the GP role could be made more attractive to newly qualified doctors and how the health service can retain its mid-career GPs. In addition, any lessons learned not only regarding future workforce planning but also a strategic approach to career development and support must be applied.
